# Trends in Longevity in the Americas: Disparities in Life Expectancy in Women and Men, 1965-2010

**DOI:** 10.1371/journal.pone.0129778

**Published:** 2015-06-19

**Authors:** Ian R. Hambleton, Christina Howitt, Selvi Jeyaseelan, Madhuvanti M. Murphy, Anselm J Hennis, Rainford Wilks, E. Nigel Harris, Marlene MacLeish, Louis Sullivan, U.S. Caribbean Alliance for Health Disparities Research Group (USCAHDR)

**Affiliations:** 1 Chronic Disease Research Centre, Tropical Medicine Research Institute, The University of the West Indies, Bridgetown, Barbados, West Indies; 2 Faculty of Medical Sciences, The University of the West Indies, Cave Hill, Barbados, West Indies; 3 Epidemiology Research Unit, Tropical Medicine Research Institute, The University of the West Indies, Kingston, Jamaica, West Indies; 4 The University of the West Indies, Kingston, Jamaica, West Indies; 5 The Sullivan Alliance, 1729 King Street, Suite 100, Alexandria, Virginia, United States of America; Institute of Psychiatry, UNITED KINGDOM

## Abstract

**Objective:**

We describe trends in life expectancy at birth (LE) and between-country LE disparities since 1965, in Latin America and the Caribbean.

**Methods & Findings:**

LE trends since 1965 are described for three geographical sub-regions: the Caribbean, Central America, and South America. LE disparities are explored using a suite of absolute and relative disparity metrics, with measurement consensus providing confidence to observed differences. LE has increased throughout Latin America and the Caribbean. Compared to the Caribbean, LE has increased by an additional 6.6 years in Central America and 4.1 years in South America. Since 1965, average reductions in between-country LE disparities were 14% (absolute disparity) and 23% (relative disparity) in the Caribbean, 55% and 51% in Central America, 55% and 52% in South America.

**Conclusions:**

LE in Latin America and the Caribbean is exceeding ‘minimum standard’ international targets, and is improving relative to the world region with the highest human longevity. The Caribbean, which had the highest LE and the lowest between-country LE disparities in Latin America and the Caribbean in 1965-70, had the lowest LE and the highest LE disparities by 2005-10. Caribbean Governments have championed a collaborative solution to the growing burden of non-communicable disease, with 15 territories signing on to the Declaration of Port of Spain, signalling regional commitment to a coordinated public-health response. The persistent LE inequity between Caribbean countries suggests that public health interventions should be tailored to individual countries to be most effective. Between- and within-country disparity monitoring for a range of health metrics should be a priority, first to guide country-level policy initiatives, then to contribute to the assessment of policy success.

## Introduction

Two cornerstones of public health policy—improving the average health of a population and reducing disparities in health [1]–imply that progress towards these goals is being actively monitored. Health monitoring is well-developed, with widely accepted methods for prospective disease surveillance [2–4], national health surveys [5], and the collection of vital statistics [6]. The associated techniques for assessing health disparities are less mature, although useful analysis techniques [7, 8] and guidelines [9–12] have been published in recent years, often adapting methodology from the economic literature [13]. Moreover, an exact definition of health disparity continues to be debated [14], remains somewhat elusive [15], but is widely linked to the concept of unfair health and healthcare differences among population subgroups due to gender, race or ethnicity, socio-economic position, geography, or other socially determined factors.

To facilitate the public health goal of monitoring and reducing disparities, the US has developed infrastructure including the Health Disparities Act (2000), which in turn allowed the creation of the National Institute on Minority Health and Health Disparities (NIMHD). The NIMHD has funded a 5-year program (NIH number: U24MD006959) to explore and compare for the first time health disparities among African-descent populations in the Caribbean and the US. This partnership between the Sullivan Alliance [16] and The University of the West Indies [17] is using published work, Caribbean health databases, and open-access data to build an evidence-based picture of Caribbean health disparities. This article is one in a series reporting disparities in a range of health domains. It is anticipated that with the creation of a comprehensive disparities situation analysis for the region, priorities for public health both regionally and on a country-level can then be based on contextually relevant evidence.

This article focuses on life expectancy at birth (LE), a basic health indicator adopted, among others, by the United Nations, the World Health Organisation, and the Organisation for Economic Co-operation and Development [18, 19]. It is a component of the Human Development Index, which itself has become a core indicator of social development and wellbeing [20], and is used widely in development planning [21, 22] and health research [23–25]. LE summaries are also readily available via a growing number of online data visualisation websites, extending its influence beyond the academic and development communities [26, 27]. Although international LE targets exist these should be considered a ‘minimum achievable standard’ [28, 29], with comparison against highest regional or country-level LE a more stringent target. The aims of this article are to describe regional LE trends and between-country LE disparities between 1965 and 2010, in Latin America and the Caribbean. This focus on describing inequities in regional LE provides an adjunct to many publicly available country-level health descriptions [30–32].

## Methods

### Data Source: United Nations World Population Prospects

Every two years the United Nations Department of Economic and Social Affairs updates its population size projections for all member countries, and includes a number of country-level demographic summaries, including LE. The World Population Projections (WPP) 2012 revision was released in June 2013 and is the source for all data used in this article [30]. As part of this effort, WPP collects mortality estimates using a variety of sources: vital registration systems, surveillance systems, demographic surveys, and censuses.

### Defining sub-regions of the Americas

In Latin America and the Caribbean, the sub-regions of Central and South America are based on well-accepted geographical boundaries. A definition for the Caribbean is less clear, and depends on historical and socio-political factors as well as geography [33]. The United Nations definition of the Caribbean includes 30 island territories in the Caribbean basin: 13 independent nations, 2 unincorporated US territories, 5 British overseas territories, 4 overseas French administrative divisions, and 6 countries or municipalities within the Kingdom of the Netherlands. Four additional territories have been added to this UN definition because of their strong historical and socio-political links to many of the Caribbean island states: Belize in Central America, and the Guianas in north-eastern South America consisting of Guyana, Suriname, and French Guiana, an overseas territory of France [34]. When considering Caribbean LE the WPP 2012 includes 21 territories with 90,000 inhabitants or more in 2012. Included and excluded Caribbean territories are listed in a Supplement to this article (S1 Table). The decision of which territories to include affects the Caribbean regional life expectancy average. In a brief sensitivity analysis, we explored how life expectancy and two measures of LE disparity (mean absolute deviation and index of disparity, each described below) changed when each of the 21 included countries were removed from the analysis, one at a time. The North American sub-region (comprising the United States of America and Canada) has been included for comparative purposes.

### Measures of health disparity

There are many potential measures of health disparity, and the properties of some of these measures have been summarized in recent reviews [8, 9, 11]. This analysis follows current pragmatic advice, presented for cancer disparity research but broadly applicable, to present a suite of disparity measures encompassing both absolute and relative measures [9]. Six disparity measures are presented: three absolute and three relative measures of disparity, with two measures considered ‘simple’ (comparing the two countries with the best and worst LE) and four considered ‘complex’ (comparing LE in all countries against a chosen comparator) according to an informal WHO classification [11]. ***The LE difference*** (LED) is an absolute disparity measure. Measured in years, it is the arithmetic difference between the two countries with the best and worst LE in each time period; in other words it reports the absolute disparity range. ***The LE ratio*** (LER) is a relative disparity measure. Measured in years, it divides the country with the best LE by that with the worst LE in each time period; in other words it reports the relative disparity range. Both measures ignore the LE experience of the countries in between these two extremes. The ***absolute mean difference*** (MD) is an absolute disparity measure. For each country it calculates the difference of its LE from the best LE in the sub-region, adds these differences together and divides by the number of countries. So MD considers all countries in the assessment group. The ***index of disparity*** (ID) [7] is a relative disparity measure. It is the absolute mean difference, expressed as a percentage of the reference country. The ***between group variance*** (BGV) is an absolute disparity measure. For each country, it calculates the squared difference of its LE from the sub-regional average, weights by the population size of the country, and adds these squared differences together. So BGV considers all countries in the assessment group, and the squared term means that BGV is sensitive to larger deviations from the sub-regional average. The ***symmetric Theil index*** (STI) is a relative summary measure. It is the average of the Theil Index and the Mean Log Deviation, and is sensitive to LE differences further from the group mean irrespective of direction [35, 36]. For LED, MD, ID, BGV and STI zero means equality and for LER one means equality. For all measures, larger positive values indicate increasing health disparity. Consensus across the suite of disparity measures would offer consistent evidence for a particular disparity trend. All measures are appropriate when there is no implicit ordering in the groups being compared, as is the case when comparing countries or world regions.

### Statistical Analysis

Sub-regional life expectancies were calculated as the weighted average of country life expectancies, with the weight being each country’s population size. LE was then plotted between 1965 and 2010 for the world, and for sub-regions of the Americas. For each year, the difference in LE was calculated compared to the world average (the LE gap) and compared to the highest sub-regional LE in each year, using 22 UN-defined world sub-regions (the LE shortfall). Next, using 5-year time periods between 1965–70 and 2005–10, the suite of six health disparity metrics were calculated: three absolute measures (LED, MD, BGV) and three relative measures (LER, ID, STI). Between-country LE disparities are the main subject of this analysis and are reported separately for women and men. All analyses were performed using Stata statistical software [37].

## Results

### Life expectancy at birth (1965–2010): regional trends in the Americas

**Fig 1:**
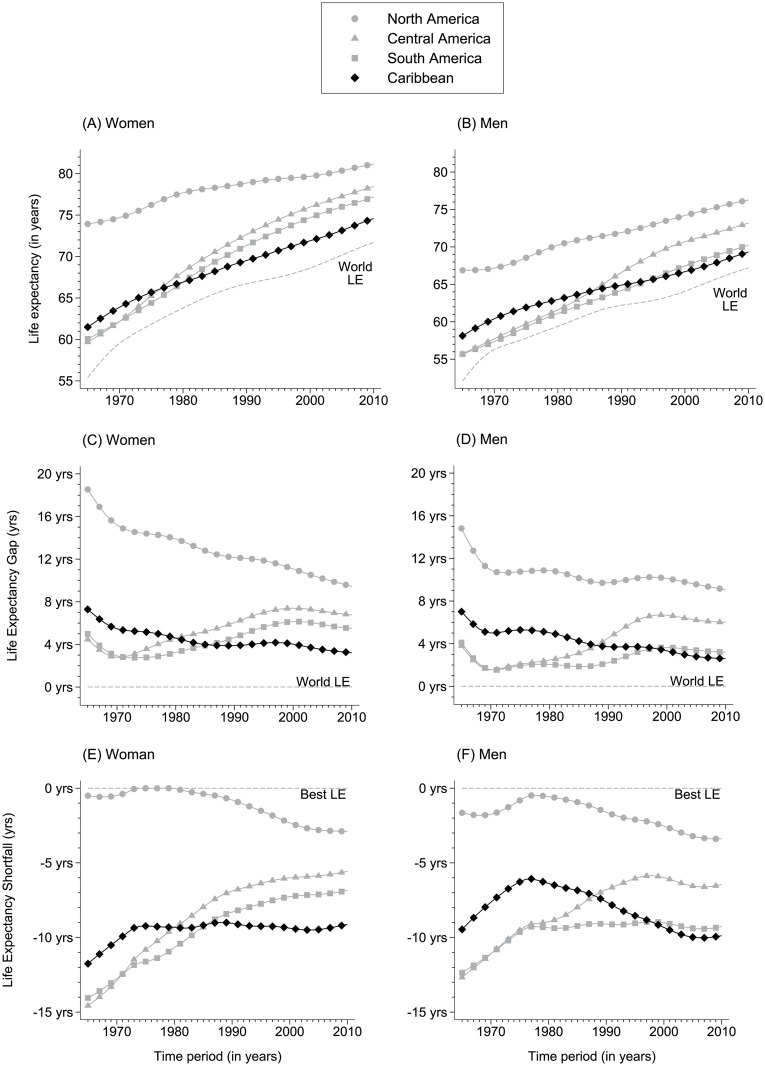
Life expectancy at birth (LE), the absolute difference in LE compared to the global average (the LE gap), and the absolute difference in LE compared to the region with the highest LE (the LE shortfall) for women and men in the Americas between 1965 and 2010.

Globally, LE rose 15.7 years to 69.4 years in the 45-years from 1965, an average annual rise of 4.2 months of life per calendar year. This consistent and striking improvement in global LE between 1965 and 2010 is repeated for each major world region and all sub-regions of the Americas. Against this positive global message, important variations exist in the region of the Americas. In 1965, LE was 57.8 years in Central America (CA), 58.2 years in SA (SA), and 60.8 years in the Caribbean, and rose during the next 45-years by 18.1 years in CA, by 15.5 years in SA, and by 11.5 years in the Caribbean (Fig 1A and 1B).

Compared to the Caribbean, LE has increased by an additional 6.6 years in CA, by an additional 4.1 years in SA, and by an additional 4.2 years globally (Fig 1C and 1D). Compared to the best sub-regional LE (which was 71.5 years in Northern Europe in 1965 and 81.9 years in Australia/New Zealand in 2010), Central and South America reduced their LE shortfall considerably; this absolute shortfall reducing from 13.7 to 6.0 years in CA and from 13.3 to 8.1 years in SA. In contrast, the Caribbean LE improvement reduced the shortfall only marginally from 10.7 years in 1965 to 9.5 years in 2010 (Fig 1E and 1F).

Considering women and men separately, the 45-year reduction in LE shortfall was generally greater in women than men, and greater in Central and South America than in the Caribbean. Among women this shortfall was reduced from 14.6 to 5.6 years in CA (a 9-year reduction), from 14.1 to 6.8 years in SA (a 7.3-year reduction), and from 11.8 to 9.1 in the Caribbean (a 2.7-year reduction). In men, it was reduced from 12.7 to 6.5 years in CA (a 6.2-year reduction), from 12.4 to 9.3 years in SA (a 3.1 year reduction), and increased from 9.5 to 9.9 in the Caribbean (a 0.4 year increase) (Fig 1E and 1F).

### Life expectancy at birth (1965–2005): gender disparities in the Americas

The global gender disparity in LE between men and women in 1965–70 was 2.9 years (4.9% difference), rising to 4.5 years (6.3% difference) in 2005–10. In Latin America and the Caribbean, women lived longer than men, with the gender difference in 2005–10 being 5.2 years (6.9% difference) in the Caribbean, 5.3 years (6.8% difference) in CA, and 6.8 years (8.9% difference) in SA (Table 1).

**Table 1:** Gender disparities in life expectancy at birth (LE) in 1965–70 and in 2005–10 among 4 sub-regions of the Americas.

Region	Year	LE (overall)	LE (women)	LE (men)	F:M Difference	% Difference	F:M Ratio
**Caribbean**	1965–70	62.08	63.95	60.34	3.62	5.65	1.06
**N = 21**	2005–10	71.79	74.42	69.26	5.16	6.93	1.07
**Central America**	1965–70	58.83	60.95	56.79	4.16	6.83	1.07
**N = 7**	2005–10	75.31	77.91	72.59	5.31	6.82	1.07
**South America**	1965–70	59.15	61.39	57.04	4.35	7.09	1.08
**N = 10**	2005–10	73.14	76.6	69.77	6.83	8.92	1.1
**North America**	1965–70	70.49	74.28	66.95	7.33	9.87	1.11
**N = 2**	2005–10	78.36	80.81	75.83	4.99	6.17	1.07
**Latin America & the Caribbean**	1965–70	58.91	61.14	56.8	4.34	7.1	1.08
**N = 38**	2005–10	73.45	76.7	70.22	6.48	8.45	1.09
**The Americas**	1965–70	64.52	67.49	61.75	5.74	8.5	1.09
**N = 40**	2005–10	75.37	78.28	72.45	5.83	7.45	1.08
**World**	1965–70	56.52	57.92	55.07	2.85	4.91	1.05
**N = 201**	2005–10	68.72	71	66.52	4.48	6.31	1.07

### Life expectancy at birth (1965–2005): between-country disparities in the Americas

Between-country LE disparities in 1965–70 and in 2005–10 are presented in Table 2 using six disparity metrics for each sub-region, separately for women and men. Globally, these disparities have decreased in the 40 years from 1965–70. On average, absolute disparity measures decreased by 19% and relative disparity measures by 33%, with reductions similar in women and men (women 17% and 31%, men 22% and 36%).

**Table 2:** Between-country disparities in life expectancy at birth (LE) in 1965–70 and in 2005–10 among 4 sub-regions of the Americas.

		Disparity measures using best and worst country LE		Disparity measures using LE from all countries			
Region	Year	LE ratio	LE difference	Mean Absolute Deviation	Index of Disparity	Symmetric Theil Index	Between Group Variation
**WOMEN ONLY**							
**Caribbean N = 21**	1965–70	1.55	26.22	8.31	11.26	33.19	26.23
	2005–10	1.33	20.81	7.10	8.53	20.86	23.46
	% change	-14.19	-20.63	-14.56	-24.25	-37.15	-10.56
**Central America N = 7**	1965–70	1.32	16.16	10.01	14.84	52.41	36.38
	2005–10	1.10	7.47	4.85	5.98	5.43	6.48
	% change	-16.67	-53.77	-51.55	-59.70	-89.64	-82.19
**South America N = 10**	1965–70	1.52	24.65	11.20	15.58	70.30	51.36
	2005–10	1.20	13.83	5.54	6.79	11.44	13.02
	% change	-21.05	-43.89	-50.54	-56.42	-83.73	-74.65
**North America N = 2**	1965–70	1.02	1.45	1.45	1.92	0.47	0.53
	2005–10	1.03	2.21	2.21	2.67	0.91	1.22
	% change	0.98	52.41	52.41	39.06	93.62	130.19
**The Americas N = 40**	1965–70	1.60	28.33	11.77	15.57	60.04	46.62
	2005–10	1.33	20.81	6.53	7.84	15.85	18.06
	% change	-16.88	-26.54	-44.52	-49.65	-73.60	-61.26
**World N = 201**	1965–70	2.42	45.05	17.44	22.72	220.01	144.85
	2005–10	1.95	41.90	14.42	16.76	115.02	108.25
	% change	-19.42	-6.99	-17.32	-26.23	-47.72	-25.27
**MEN ONLY**							
**Caribbean N = 21**	1965–70	1.51	22.75	6.41	9.47	34.45	24.02
	2005–10	1.30	17.75	6.28	8.19	19.43	18.78
	% change	-13.91	-21.98	-2.03	-13.52	-43.60	-21.82
**Central America N = 7**	1965–70	1.30	14.91	10.15	15.88	57.95	35.92
	2005–10	1.15	10.00	6.44	8.42	12.12	12.22
	% change	-11.54	-32.93	-36.55	-46.98	-79.09	-65.98
**South America N = 10**	1965–70	1.53	22.55	9.10	13.90	66.96	41.28
	2005–10	1.19	12.06	5.60	7.42	9.12	8.88
	% change	-22.22	-46.52	-38.46	-46.62	-86.38	-78.49
**North America N = 2**	1965–70	1.03	2.19	2.19	3.18	1.30	1.20
	2005–10	1.03	2.60	2.60	3.33	1.43	1.69
	% change	0.00	18.72	18.72	4.72	10.00	40.83
**The Americas N = 40**	1965–70	1.61	25.99	9.47	13.73	58.71	39.40
	2005–10	1.33	19.22	7.36	9.41	16.41	16.15
	% change	-17.39	-26.05	-22.28	-31.46	-72.05	-59.01
**World N = 201**	1965–70	2.40	41.84	16.53	23.03	202.39	115.50
	2005–10	1.82	35.75	12.73	16.00	97.35	81.56
	% change	-24.17	-14.56	-22.99	-30.53	-51.90	-29.39
**WOMEN & MEN**							
**Caribbean N = 21**	1965–70	1.53	24.47	7.31	10.33	33.41	24.78
	2005–10	1.32	19.43	6.87	8.58	20.16	21.04
	% change	-13.73	-20.6	-6.02	-16.94	-39.66	-15.09
**Central America N = 7**	1965–70	1.31	15.49	10.05	15.32	54.3	35.6
	2005–10	1.12	8.52	5.58	7.09	7.79	8.57
	% change	-14.5	-45	-44.48	-53.72	-85.65	-75.93
**South America N = 10**	1965–70	1.52	23.5	10.06	14.67	67.85	45.47
	2005–10	1.2	13	5.64	7.18	10.18	10.73
	% change	-21.05	-44.68	-43.94	-51.06	-85	-76.4
**North America N = 2**	1965–70	1.02	1.74	1.74	2.41	0.74	0.76
	2005–10	1.03	2.41	2.41	2.99	1.15	1.45
	% change	0.98	38.51	38.51	24.07	55.41	90.79
**The Americas N = 40**	1965–70	1.6	27.02	10.44	14.48	58.87	42.46
	2005–10	1.33	19.86	6.8	8.45	15.92	16.88
	% change	-16.87	-26.5	-34.87	-41.64	-72.96	-60.24
**World N = 201**	1965–70	2.4	43.26	16.78	22.66	210.91	129.39
	2005–10	1.88	38.7	13.48	16.3	104.9	93.15
	% change	-21.67	-10.54	-19.67	-28.07	-50.26	-28.01

Average between-country LE disparities reduced consistently between 1965–70 and 2005–10 in each sub-region of Latin America and the Caribbean, with Latin American reductions substantially greater than those in the Caribbean. In Central America, absolute disparities reduced by 55% and relative disparities by 51%. In SA the reductions were 55% and 52%, and in the Caribbean they were 14% and 23%. Reductions were broadly similar in women and men in each sub-region. Among women, absolute and relative disparity reductions were 63% and 55% in CA, 56% and 54% in SA and 15% and 25% in the Caribbean. Equivalent disparity reductions among males were 45% and 46% in CA, 54% and 52% in SA, 15% and 24% in the Caribbean.

**Fig 2:**
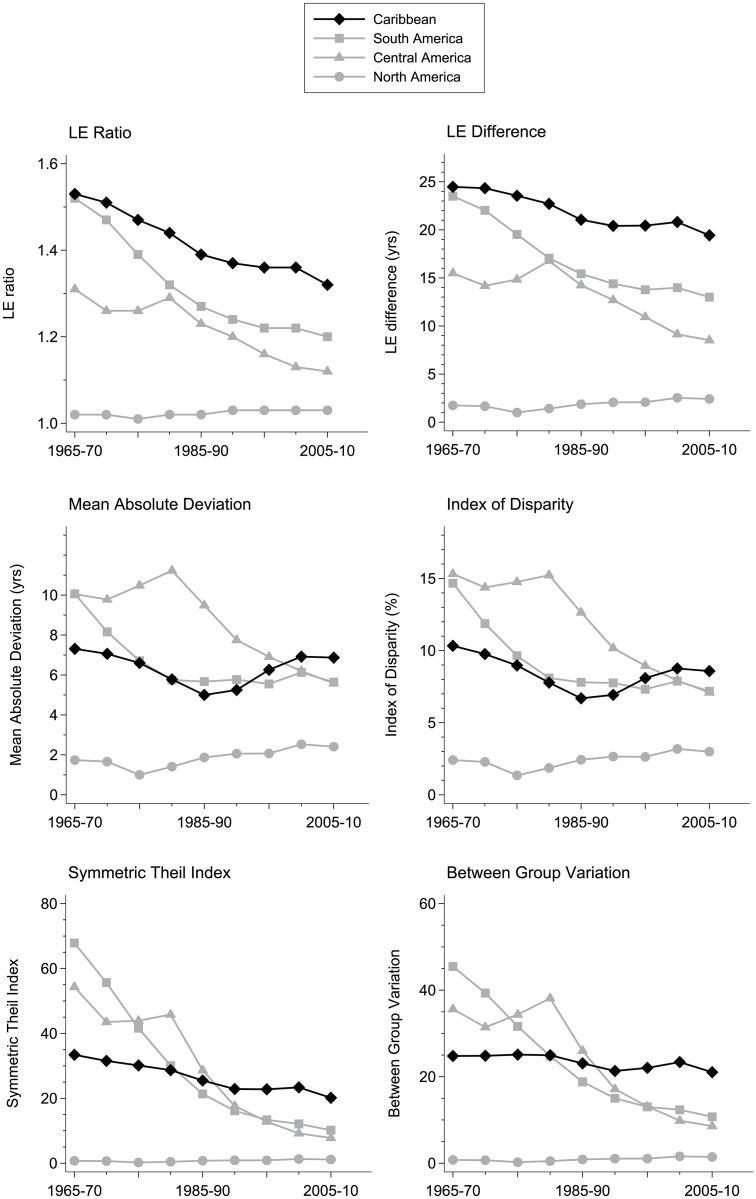
Trends in between-country disparities using two ‘simple’ disparity metrics (LER, LED), and four ‘complex’ disparity metrics (MD, ID, STI, BGV) for four sub-regions of the Americas, 1965–2010 (women and men combined).

LE disparity trends between 1965–70 and 2005–10 are plotted in Fig 2 (for indices using best and worst country life expectancies, LER and LED, and for indices using all country life expectancies, MD, ID, STI, BGV. Disparity levels using LER and LED, which consider just the extremes of the country LE distribution, are always lowest in North America and are always highest in the Caribbean. The low North American disparity is unsurprising as the sub-region includes two industrialised nations of the USA and Canada, while the high Caribbean rate is influenced in part by Haiti, with its consistently low LE. Disparity levels using MD, ID, STI and BGV each highlight modest Caribbean disparity reductions compared to larger Latin American reductions, leaving the Caribbean as the sub-region with the highest absolute and relative disparity levels by 2005–10.

### Sensitivity analysis: changing Caribbean-region membership

The Caribbean-wide (N = 21 countries) life expectancy for women and men combined was 62.1 years in 1965–70 and 71.8 years in 2005–10, an absolute increase of 9.7 years and a percentage increase of 15.7%. We removed 1 Caribbean country at a time to create twenty-one different 20-country groupings. The results of this sensitivity analysis are presented in a Supplement to this article (S2 Table). Among these alternative regional groupings, the average life expectancy at birth in 1965–70 was between 58.9 years (removing Cuba) and 65.5 years (removing Haiti), and in 2005–10 was between 69.4 years (removing Cuba) and 75.0 years (removing Haiti). Despite these large variations in absolute LE, varying the country-membership of the Caribbean region had little effect on the LE change between 1965–70 and 2005–10: LE increased by between 8.6 years (13.6%) when removing Dominican Republic and 10.5 years (17.8%) when removing Cuba. This compared to LE increases of 16.5 years (28.0%) in Central America and 14.0 years (23.7%) in South America. The change in LE disparities between 1965–70 and 2005–10 was also less affected by changing Caribbean-region membership. The Caribbean-wide (N = 21 countries) reductions in the index of disparity (ID) were 16.9% in the Caribbean, 53.7% in Central America and 51.1% in South America. When we varied our Caribbean regional membership, ID reductions varied between 13.2% (removing Guadeloupe) and 21.8% (removing Martinique), with the ID increasing by 14% if Puerto Rico were removed from the Caribbean regional definition.

Cuba and Haiti, as the largest countries in the Caribbean region, might have been expected to heavily influence results. We paid particular attention to these two countries during this sensitivity work. Haiti had a population of approximately 9.3 million in 2005–10, about 22% of the 21 country Caribbean population. It had a LE of 60.7 years in 2005–10, 19.4 years below Martinique as the best performing Caribbean country. Excluding Haiti increased the regional LE average by 3.2 years, to be 1.8 years higher than South America and 0.3 years lower than Central America in 2005–10. Its removal had little impact on the percentage increase in LE since 1965–70 (15.6% increase with Haiti, 14.5% increase without Haiti), and had little impact on the percentage decrease in regional disparities (Index of disparity: 17.0% decrease with Haiti, 14.4% decrease without Haiti). So although the removal of Haiti from a definition of the Caribbean pushed up regional average life expectancy, it did not materially affect LE change or LE disparity change over time. Since 1965–70, the Caribbean has underperformed compared to its regional neighbours, with or without Haiti. Cuba had a population of approximately 11.3 million in 2005–10, about 27% of the 21 country Caribbean population. It had a LE of 78.3 years in 2005–10, 1.8 years below Martinique as the best performing Caribbean country. Conversely, excluding Cuba decreased the regional LE average by 2.4 years, to be 3.8 years lower than South America and 5.9 years lower than Central America in 2005–10. Again, its removal had little impact on the percentage increase in LE since 1965–70 (15.6% increase with Cuba, 17.8% increase without Cuba), and had little impact on the percentage decrease in regional disparities (Index of disparity: 17.0% decrease with Cuba, 16.8% decrease without Cuba). So removing Cuba from a definition of the Caribbean pushed down regional average life expectancy, but again did not materially affect LE change or LE disparity change over time. Since 1965–70, the Caribbean has underperformed compared to its regional neighbours, with or without Cuba.

## Discussion

### Life expectancy disparities across the Americas

In the Americas, LE has increased consistently since 1965 and 33/38 countries (87%) now report a LE above 70 years: all countries in Central America, 9/10 in South America and 17/21 in the Caribbean. These improvements are a fundamental public health success. However, reviewing the data with a focus on regional disparities in LE presents a more nuanced picture, with Latin America able to claim greater relative success compared to the Caribbean. In Latin America and the Caribbean, 45-year gains in LE ranged from 4.9 to 18.0 years among Caribbean countries, from 7.2 to 23.7 years in South America, and from 13.7 to 23.5 years in Central America, so that the Caribbean, which had the highest LE in Latin America and the Caribbean in 1965, had the lowest LE by 1987. This Caribbean-Latin American LE difference has continued to widen thereafter. The Latin American sub-regions have reduced their LE shortfall compared to the best performing sub-region, whereas the Caribbean has not. Reductions in between-country disparities in LE at birth have also been enjoyed throughout the Americas, with larger reductions seen in Central and South America compared to the Caribbean. Average reductions in absolute and relative disparities in the Caribbean between 1965–70 and 2005–10 were 14% and 23%, in CA the reductions were 55% and 51%, and in SA they were 55% and 52%. These larger Latin American reductions have left the Caribbean as the sub region with the largest between-country disparities in LE, irrespective of the disparity metric used.

### Monitoring national health using LE

LE at birth is a measure of health and social development used widely by international agencies [19]. Recognising that international LE targets should be seen as a ‘minimum achievable standard’, the Declaration of Alma Ata (1978) set a LE target of 60 years by the year 2000. Of the 47 countries not reaching this target, 44 were in Africa. The 1994 International Conference on Population and Development (ICPD) set new LE targets of 70 years or more by 2005 and 75 years or more by 2015. The ICPD targets recognised existing LE disparities by reducing the targets to 65 and 70 years “for countries with the highest levels of mortality” [28]. Recognising the monitoring ambiguity introduced by this mortality clause, around one quarter of countries had not reached a LE of 65 years by 2005–10, and over 40% had not achieved the more stringent target of 70 years. In the Caribbean, four countries—Guyana, Haiti, Suriname, Trinidad & Tobago—had not achieved an overall LE of 70 years with just one country—Haiti—not achieving 65 years.

### Public health implications of LE disparities

The ICPD-at-15 reviewed its wide-ranging goals and had this to say about Caribbean LE: *“In the English- and Dutch-speaking Caribbean*, *population growth has dropped to around replacement levels and life expectancy is relatively high*.*”* [29]. A positive picture indeed, and an accurate one. Nevertheless, the statement masks within-region LE disparities, which are important to regional and country-level policy planning initiatives such as the Caribbean Cooperation in Health (CCH) and the Declaration of Port-of-Spain. Adopted by the 15 member states of the Caribbean Community (CARICOM) in 1984, and now in its third phase (2010–2015), the CCH has the overarching goal of “improving and sustaining the health of the people of the Caribbean, adding years to life and life to years” [38]. The Caribbean has for some time accepted the role of non-communicable diseases (NCDs) on regional health. The Declaration of Port-of-Spain, issued in 2007, calls for unity amongst Caribbean Community member states in the response to NCDs. We have highlighted persistent LE differences between Caribbean countries, and this intractable inequity complicates the regional commitment to a coordinated public-health response [39, 40], suggesting that public health interventions should be tailored to individual countries to be most effective.

The NCD burden poses new governance challenges: the causes are multifactorial, the affected populations diffuse, and effective responses require sustained multi-sectorial cooperation. The relative contribution of lifestyle changes and medical intervention has been explored [41], with a general recognition that solutions will be costly, lengthy, and will rely partly on regulatory change [42, 43].

The Caribbean is dominated by Small Island Developing States (SIDS), and this fact may make the NCD response harder to sustain. The United Nations currently recognises 51 SIDS, with 22 of these in the Caribbean, and 17 in our 21-country Caribbean grouping [44]. Although diverse in terms of economic development, they are especially vulnerable to economic shocks and external influences and this is reflected in the lower Caribbean annual growth and greater volatility in that growth compared to Central and South America [45]. The Economic Vulnerability Index (EVI), calculated every 3-years since 2006 by the UN, always rates the Caribbean as more vulnerable than its Latin American neighbours. In 2006, average EVI was 50.6 in the Caribbean, compared to 34.0 in CA and 37.0 in SA [46]. Efforts are ongoing to improve resilience; average vulnerability in 2015 dropped to 38.3 in the Caribbean, 26.7 in CA, and 30.0 in SA, with the UN-backed SAMOA pathway now recognising the need for an NCD response among SIDS, and also accepting that this response will require meaningful collaboration between national and international partners [47].

Monitoring is needed to accompany these public health efforts, and lessons from the Millennium Development Goal (MDG) era must inform NCD data collection and reporting. MDG monitoring regularly suffered from data that was old, incomplete and of poor quality, with insufficient investment in the strengthening of national statistical capacity [48]. Moreover, MDG monitoring focused on average change, whereas reporting equity-adjusted measures is now considered by many to be central to national health monitoring [49].

### Monitoring implications of LE disparities

This NIH-funded project represents a new facet of the regional response to monitoring and improving health and reducing health disparities. We propose that LE as a ‘performance metric’ should be supplemented by measures of health variation at two levels of reporting.

First, between-country disparity measures could allow decision makers to assess national performance alongside their Caribbean neighbours, who will be subject to many of the same external international influences, but perhaps be pursuing different public health programmes. Between-country disparity monitoring of LE should include as a minimum the LE ‘shortfall’ comparing the region and each country to that world region or country with the highest LE. A suite of between-country disparity measures, as we have provided in this report, should be tracked through time, and free software is available to ease the analytical burden of these efforts [50].

Second, within-country disparity measures should allow decision makers to track disparities between key population subgroups through time, and so become core monitoring tools for the evaluation of sub-national public health interventions. Central to such monitoring is the acceptance that as a country achieves national health targets, vulnerable minority groups with poorer health outcomes will persist. A focus for within-country disparities monitoring should be the active reporting of socially determined population subgroups, stratified for example by gender, by socio-economic position, by geography, or by race/ethnicity. The WHO STEPS surveys, conducted throughout the Caribbean, is one possible data source for such within-country monitoring [51].

### Between-country and between-gender disparities in life expectancy

This article reports on two social determinants of health: place of residence and gender. They have been identified as two of eight key drivers of health inequity: place of residence, race or ethnicity, occupation, gender, religion, education, socioeconomic position, and social capital—the so-called “progress” acronym [11, 52, 53]. Between-country disparities in LE are socially determined and reflect a true health inequity, insofar as the LE shortfall for any given country can be seen as an unnecessary and so unfair disparity. This between-country disparity, reported at the sub-regional level, is the main subject of this article. In keeping with existing research on gender disparities in health, women have a higher LE then men [54]. Although LE differences *between* women and men are also strongly influenced by social factors (gender differences), there is good evidence that innate biological factors also confer a female biological advantage (sex differences), and that these social and biological factors may interact, making a target disparity between women and men difficult to promote [55].

### Study Limitations: data quality

The completeness of mortality data collected by a country’s vital registration system is an important determinant of LE validity, and indicators of data quality are reported in the United Nations World Mortality Report [56] and more fully in two reports investigating metrics of vital registration quality [57, 58]. Although the UN Statistics Division and the World Health Organization are the agencies primarily responsible for compiling and disseminating international fertility and mortality data, they are dependent on annual submissions from member countries, and without reliable national data must resort to LE estimation. A better understanding of health metric data quality in the Americas should be a key analytical priority. When using information derived from vital registration data, it will be important to report clearly the methods used and assumptions made to accommodate data imperfections, and sensible sensitivity analyses will be key to understanding the practical importance of these adjustment methods and assumptions. A second source of country-level life expectancy estimates is the Global Burden of Disease Study (GBD) [59]. Although using many of the same data sources, the recent GBD estimates can differ from WPP estimates, occasionally substantially so [60]. With both estimation efforts based on robust demographic and statistical principles, country-level differences require further exploration, with an important question being whether choice of estimate fundamentally changes public health messaging.

### Selecting metrics for monitoring disparities

The choice of disparity summary measure is important, and three considerations should help to guide this choice: whether to report relative or absolute disparity, how to examine departures from health equality (in other words what is an appropriate reference group), and whether to weight the disparity measure by the size of each population sub-group? A fourth consideration, that the concept of “health disparity” remains conceptually ambiguous, has fundamental importance for indicator choice. It has been recognised that if our understanding of disparity remains ambiguous, then an accurate measure of disparity should preserve this ambiguity [61]. In practice this means that different health disparity summaries measure different aspects of inequality. Rather than choosing an individual measure, this analysis presents a suite of disparity measures encompassing both absolute and relative measures [9]. This paper therefore implicitly includes a sensitivity analysis to test the robustness of conclusions from any single disparity metric. The measures in this article are appropriate when there is no ordering in the groups being compared, as is the case when comparing countries or world regions [9, 11]. Other metrics will be suited to comparing groups with an implicit ordering (such as comparisons based on level of education, or income).

## Conclusion

Since 1965, against a backdrop of important LE improvements throughout the Americas, LE in the Caribbean has increased less quickly than in Latin America, with disparities in Caribbean LE barely changed. This relative performance has left the Caribbean as the sub-region of the Americas with the lowest LE, and the largest between-country disparities in LE, irrespective of the disparity metric used. Caribbean Governments have championed a collaborative solution to the growing burden of non-communicable disease, with 15 territories signing on to the Declaration of Port of Spain, signalling regional commitment to a coordinated public-health response [39]. The persistent LE inequity between Caribbean countries suggests that public health interventions should be tailored to individual countries to be most effective, making the commencement of between- and within-country disparity monitoring for a range of health metrics a priority, first to guide country-level policy initiatives, then to contribute to the assessment of policy success.

## Supporting Information

S1 Table Countries and territories included in definition of the Caribbean, with estimated population in 2012. (XLS)

S2 Table Sensitivity analysis: Change in life expectancy at birth and two measures of disparity (mean absolute deviation and index of disparity) between 1965–70 and 2005–10 when removing 1 country at a time from the 21-country Caribbean regional grouping. (XLS)
